# The Role of Dietary Fibre in Modulating Gut Microbiota Dysbiosis in Patients with Type 2 Diabetes: A Systematic Review and Meta-Analysis of Randomised Controlled Trials

**DOI:** 10.3390/nu12113239

**Published:** 2020-10-23

**Authors:** Omorogieva Ojo, Qian-Qian Feng, Osarhumwese Osaretin Ojo, Xiao-Hua Wang

**Affiliations:** 1 School of Health Sciences, Faculty of Education, Health and Human Sciences, University of Greenwich, Avery Hill Campus, Avery Hill Road, London SE9 2UG, UK; 2 The School of Nursing, Soochow University, Suzhou 215006, China; 20195231027@stu.suda.edu.cn (Q.-q.F.); wangxiaohua@suda.edu.cn (X.-h.W.); 3 South London and Maudsley NHS Foundation Trust, University Hospital, Lewisham High Street, London SE13 6LH, UK; Osarhumwese.Ojo@slam.nhs.uk

**Keywords:** type 2 diabetes, dietary fibre, gut microbiota, dysbiosis, short-chain fatty acids, glycated haemoglobin, fasting blood glucose, adverse events

## Abstract

Background: The prevalence of type 2 diabetes is on the increase worldwide, and it represents about 90% of adults who are diagnosed with diabetes. Overweight and obesity, lifestyle, genetic predisposition and gut microbiota dysbiosis have been implicated as possible risk factors in the development of type 2 diabetes. In particular, low intake of dietary fibre and consumption of foods high in fat and sugar, which are common in western lifestyle, have been reported to contribute to the depletion of specific bacterial taxa. Therefore, it is possible that intake of high dietary fibre may alter the environment in the gut and provide the needed substrate for microbial bloom. Aim: The current review is a systematic review and meta-analysis which evaluated the role of dietary fibre in modulating gut microbiota dysbiosis in patients with type 2 diabetes. Methods: This is a systematic review and meta-analysis of randomised controlled trials which relied on the Preferred Reporting Items for Systematic Reviews and Meta-Analyses (PRISMA) framework. Electronic searches were conducted using EBSCOHost with links to Health Sciences Research Databases, EMBASE and Google Scholar. The reference lists of articles were also searched for relevant studies. Searches were conducted from date of commencement of the database to 5 August 2020. The search strategy was based on the Population, Intervention, Comparator, Outcomes, Studies (PICOS) framework and involved the use of synonyms and medical subject headings (MesH). Search terms were combined with Boolean operators (OR/AND). Results: Nine studies which met the inclusion criteria were selected for the systematic review and meta-analysis, and four distinct areas were identified: the effect of dietary fibre on gut microbiota; the role of dietary fibre on short-chain fatty acids (SCFAs); glycaemic control; and adverse events. There was significant difference (*p* < 0.01) in the relative abundance of Bifidobacterium with a mean difference of 0.72 (95% CI, 0.56, 0.89) between the dietary fibre group compared with placebo. In relation to the meta-analysis for SCFAs, while there was significant difference (*p* = 0.02) between the dietary fibre group and placebo with a standardised mean difference of 0.5 (95% CI, 0.08, 0.91) regarding total SCFAs, the differences were not significant (*p* > 0.05) in relation to acetic acid, propionic acid and butyric acid. There was only significant improvement (*p* = 0.002) with respect to glycated haemoglobin with a mean difference of −0.18 (95% CI, −0.29, −0.06) between the dietary fibre group and placebo group. Differences between the two groups were not significant (*p* > 0.05) in relation to fasting blood glucose and homeostatic model assessment of insulin resistance (HOMA-IR). Furthermore, there were no significant differences between the two groups in subjects who reported adverse events. It is possible that the promotion of SCFA producers in greater diversity and abundance by dietary fibre in this review led to improvement in glycated haemoglobin, partly due to increased glucagon-like peptide-1 (GLP-1) production. In addition, Bifidobacterium lactis has been reported to increase glycogen synthesis, decrease expression of hepatic gluconeogenesis genes, improve translocation of glucose transport-4 and promote glucose uptake. It is also possible that the reduction in body weight of participants in the intervention group compared with control may have contributed to the observed improvement in glycated haemoglobin. Conclusion: This systematic review and meta-analysis have demonstrated that dietary fibre can significantly improve (*p* < 0.05) the relative abundance of Bifidobacterium, total SCFAs and glycated haemoglobin. However, dietary fibre did not appear to have significant effect (*p* > 0.05) on fasting blood glucose, HOMA-IR, acetic acid, propionic acid, butyric acid and adverse events.

## 1. Introduction

The prevalence of type 2 diabetes is on the increase globally, and it represents about 90% of adults who are diagnosed with diabetes [1,2]. The World Health Organisation [3] has reported an increase from an estimated 108 million in 1980 to 422 million adults living with type 2 diabetes in 2014. The prevalence of type 2 diabetes is predicted to reach 642 million by the year 2040 [4]. Diabetes as a condition can have significant impact on mortality and morbidity including acute and long-term complications [5]. There is evidence that diabetes is a major risk factor in the development of kidney dysfunction, retinopathy, neuropathy and cardiovascular diseases [6]. Overweight and obesity, lifestyle, genetic predisposition and gut microbiota dysbiosis have been implicated as possible risk factors in the development of type 2 diabetes [6]. For example, low intake of dietary fibres and intake of foods high in fat and sugar, which are common in western lifestyle, have been reported to contribute to the depletion in the abundance of specific bacterial taxa and the diversity of gut microbial community [7]. It is possible that gut microbiota dysbiosis may influence the metabolic and functional pathways in the gut which are involved in the development of type 2 diabetes [6].

## 2. Description of the Intervention

An understanding of the pathophysiology of type 2 diabetes and the various management approaches is crucial in reducing the risks of diabetes and its complications. In particular, the use of dietary interventions including dietary fibre has been promoted by researchers and practitioners involved in diabetes care and management. However, there appears to be inconsistencies in what is considered to be a dietary fibre [8]. In 2008, the Scientific Advisory Committee on Nutrition (SACN) [9] defined dietary fibre as non-starch polysaccharides (NSP). The definition of dietary fibre was revised in 2015 to include all carbohydrates that are neither digested nor absorbed in the small intestine and have some degree of polymerisation of three or more monomeric units and lignin [10]. Thus, the components of dietary fibre include total fibre, NSP, fibre components from cereal, fruits and vegetables, polydextrose, oligosaccharides (including fructo-oligosaccharides, galacto-oligosaccharides and inulin), lignin and soluble fibres (including pectin and guar gum) [8,9,10]. According to Makki et al. [7], dietary fibre can be subdivided into polysaccharides (NSP), resistant starch, and resistant oligosaccharides. The subdivision can also be in the form of insoluble and soluble fibres [7].

## 3. How the Intervention Might Work

There is evidence that diets play a significant role in modulating gut microbiota in terms of its composition and in the production of short-chain fatty acids (SCFAs) [11]. However, our current understanding of the exact relationships between the human gut microbiome and disease remains limited [12]. It has been reported that the carbohydrates that are not digested and absorbed in the small intestine undergo fermentation by the community of commensal bacteria in the colon, which results in the formation of SCFAs, some of which are absorbed into the blood and used as sources of energy [10,13]. The SCFAs are mainly acetic acid, propionic acid and butyric acid, and they also regulate the host metabolism and inflammation [14]. SCFAs stimulate the secretion of gastric inhibitory polypeptide (GIP), glucagon-like peptide-1 (GLP-1) and peptide YY (PYY) in adipocytes, leading to reduced fat accumulation [14]. In addition, the micro-organisms that reside in the gastrointestinal tract have been implicated in health and disease [11]. In relation to bacterial taxonomy of the human gut microbiota, the four main phyla are Actinobacteria, Bacteroidetes, Firmicutes and Proteobacteria, and these are essential for the host metabolism and physiological regulation [2,6,15]. The healthy balance of the microbes in the gut (eubiosis) may be disrupted, leading to imbalance or impaired gut microbiota distribution (dysbiosis), which have been shown to contribute to insulin resistance in type 2 diabetes [6,7]. For example, many factors such as diet, lifestyle, gut permeability have been known to affect the composition of the gut microbiota [6].

Poor dietary habits can lead to intestinal dysbiosis, such as alterations in the balance of the different bacterial phyla, including overgrowth of Proteobacteria and/or reduction in Bacteroidetes [16]. There is evidence that the proliferation of some bacteria species of the Proteobacteria phylum may lead to energy disequilibrium among the different bacteria species, suppression of the growth of other bacteria species and the development of diseases, including type 2 diabetes [16]. Therefore, the consumption of high dietary fibre may alter the environment in the gut and provide the needed substrate for microbial growth and proliferation, and the production of SCFAs [7]. SCFAs are essential sources of energy and are involved in regulating host metabolism, immune system and the proliferation of cells [7]. SCFAs can be used as sources of energy in the colonocytes, but can also be transported to the peripheral circulation through the portal vein to the liver and peripheral tissues. Therefore, poor dietary fibre consumption may not only reduce bacteria diversity in the gut, but also reduces SCFA production and a shift towards the utilisation of less favourable substrates, such as dietary and endogenous protein sources, by the gut microbiota [7]. The fermentation of proteins and amino acids by the microorganisms in the gut can lead to reduced production of total SCFAs and butyrate as well as increased production of cytotoxic and proinflammatory metabolites that contribute to the development of chronic diseases, including type 2 diabetes [7].

## 4. Why It Is Important to Do This Review

Although an association between higher intake of dietary fibre and reduced incidence of type 2 diabetes has been indicated, the results from different studies are not consistent [10]. Furthermore, studies have revealed that non-digestible oligosaccharides may affect the community of bacteria that make up the gut microflora, and their effect on health is an evolving area of research [10]. Previous systematic reviews on gut microbiota and type 2 diabetes have focused on the broad subjects of dietary and/or lifestyle interventions [17] or probiotics and faecal microbial transplantation [6] and not primarily on dietary fibre. To our knowledge, we have not found any meta-analysis on the effect of dietary fibre on gut microbiota in patients with type 2 diabetes.

### 4.1. Aim

The current review is a systematic review and meta-analysis which evaluated the role of dietary fibre in modulating gut microbiota dysbiosis in patients with type 2 diabetes.

### 4.2. Methods

This is a systematic review and meta-analysis of randomised controlled trials which relied on the Preferred Reporting Items for Systematic Reviews and Meta-Analyses (PRISMA) framework [18].

### 4.3. Types of Studies

Only randomised controlled studies were included in this review.

### 4.4. Types of Participants

The participants were people with type 2 diabetes, or in some studies, the control subjects did not have type 2 diabetes.

### 4.5. Types of Interventions

The interventions were dietary fibre including a macrobiotic diet.

### 4.6. Types of Outcome Measures

The following were the outcome measures of interest:

Relative abundance of gut microbiota (genera only): Bifidobacterium, Lactobacillus, Roseburia, Bacteroides, Ruminococcus, and Clostridium.

Short-chain fatty acids (SCFAs): total SCFA, acetic acid, propionic acid, butyric acid.

Glycaemic parameters: glycated haemoglobin (HbA1c), fasting blood glucose (FBG), homeostatic model assessment of insulin resistance (HOMA-IR).

Adverse events: total adverse events, diarrhoea, bloating, constipation, abdominal pain.

### 4.7. Search Methods for Identification of Studies

Electronic searches were conducted using EBSCOHost with links to Health Sciences Research Databases (encompassing Academic Search Premier, MEDLINE, Psychology and Behavioral Sciences Collection, APA PsycInfo, CINAHL Plus with Full Text and APA PsycArticles databases). Other electronic databases searched were EMBASE and Google Scholar. The reference lists of articles were also searched for relevant studies. Searches were conducted from date of commencement of database to 5 August 2020. The search strategy was based on the Population, Intervention, Comparator, Outcomes, Studies (PICOS) framework [19] involving the use of synonyms and medical subject headings (MesH) (Table 1). Search terms were combined with Boolean operators (OR/AND). The searches were conducted independently by two researchers (OO; OOO) and cross checked by the fourth researcher (X.W.). Differences were resolved through consensus. Articles retrieved through the electronic database searches were exported to EndNote (Analytics, Philadelphia, PA, USA) to remove the duplicates.

### 4.8. Data Collection and Analysis

#### 4.8.1. Selection of Studies

**Figure 1:**
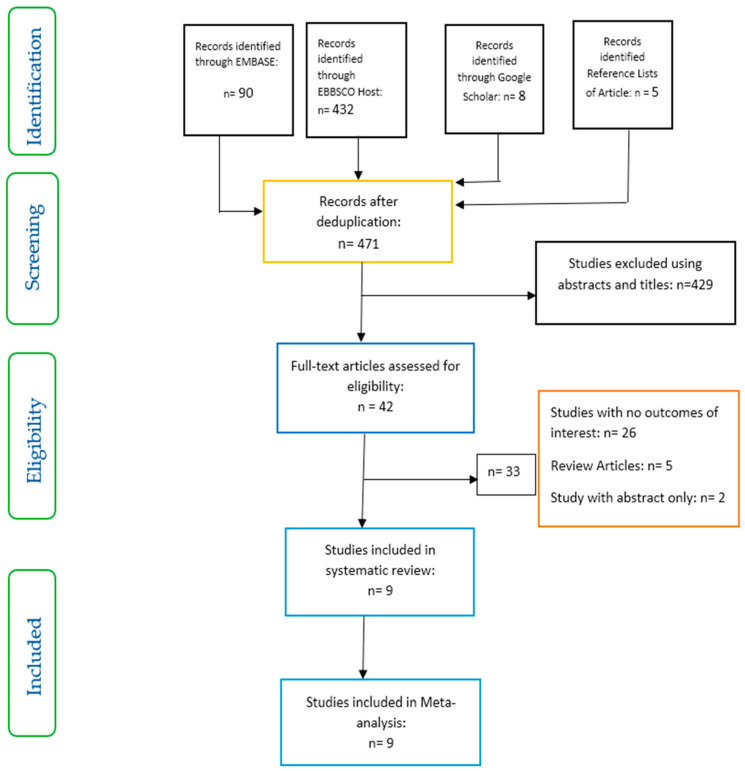
Preferred Reporting Items for Systematic Reviews and Meta-Analyses (PRISMA) flow chart on selection and inclusion of studies.

Studies were included based on a set of inclusion and exclusion criteria and relied on the PRISMA flow chart (Figure 1).

Inclusion criteria: studies selected were those involving patients with type 2 diabetes (in some studies, patients without type 2 diabetes were used as control, however, the focus in these studies was on the intervention involving only patients with type 2 diabetes); above 18 years of age; dietary fibre as intervention; and gut microbiota, glycaemic parameters, short-chain fatty acids and adverse events as outcomes of interest.

Exclusion criteria: studies involving participants below 18 years of age, patients with type 1 diabetes, and pre-diabetes or gestational diabetes were excluded. Furthermore, studies involving animal models and probiotics were also excluded.

#### 4.8.2. Data Extraction and Management

Data from the selected articles were extracted by two researchers (X.W.; Q-Q.F.) and cross-checked by the other two researchers (OO; OOO). For the meta-analysis data, the authors of the selected articles were contacted for the original data where possible. Changes from baseline for the intervention were compared with the control in all the parameters analysed [20]. The Engauge Digitizer [21] was used to extract data for the genus Bifidobacterium from the graphs in the studies of Medina-vera et al. [22] and Pedersen et al [23]. Units of measurements were converted to mmol/L for fasting blood glucose and percentage (%) for glycated haemoglobin and relative abundance of Bifidobacterium, as necessary. Furthermore, median and 1st–3rd quartiles were converted to means and standard deviations, respectively.

#### 4.8.3. Assessment of Risk of Bias and Quality of Included Studies

The risk of bias for the included studies was assessed using a domain based assessment tool [20]. The domains evaluated included the random sequence generation (selection bias), allocation concealment (selection bias), blinding of participants and personnel (performance bias), blinding of outcome assessment (detection bias), incomplete outcome data (attrition bias), selective reporting (reporting bias), and other bias [20]. The process was carried out using Review Manager 5.3 software [24]. In addition, the Critical Appraisal Skills Programme [25] checklist for randomised controlled trials was used to assess the quality of the included articles.

#### 4.8.4. Data Analysis

The meta-analysis was carried out using Review Manager (RevMan) 5.3 software [24]. In addition, sensitivity analysis was conducted by removing one study at a time from the meta-analysis in order to assess the level of consistency of the results. The assessment of heterogeneity was by means of *I*^2^ statistic [20], and *p <* 0.10 was taken as the level of statistical significance of heterogeneity. The fixed effects model and mean difference were used for the meta-analysis. However, for short-chain fatty acids data, these were converted into standardised mean difference (SMD) due to the use of different measurement scales in the outcome of interest and the random effects model was used for the analysis.

#### 4.8.5. Effect Size

The overall effect of the intervention in relation to statistical significance was based on *p* < 0.05, and the results of the meta-analysis were presented as forest plots.

## 5. Results

Nine studies which met the inclusion criteria were selected for the systematic review and meta-analysis (Figure 1). The characteristics of the studies included are shown in Table 2. While three of these studies were conducted in Italy [26,27,28], one study each was carried out in Norway [29], Japan [30], Mexico [22], UK [23], Canada [31] and China [13]. All the studies were randomised controlled trials with parallel design, except one which was a randomised cross-over study [29].

### 5.1. Risk of Bias in Included Studies

**Figure 2:**
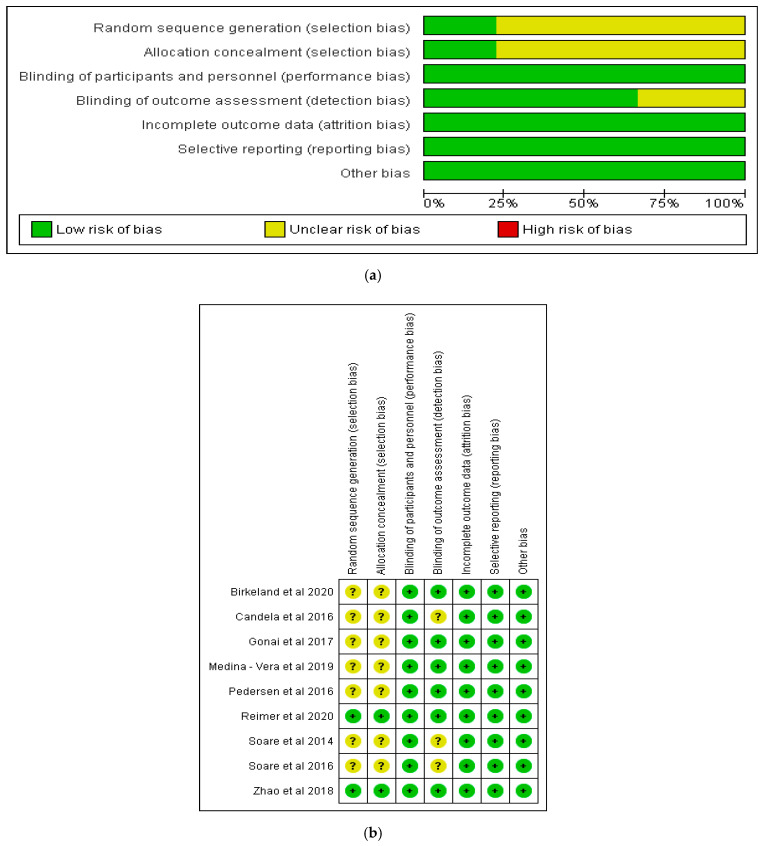
(**a**) Risk of bias graph for the included studies. (**b**) Risk of bias summary for the included studies.

The risks of bias in the included studies are shown in Figure 2a,b. Of the studies, 100% showed a low risk of bias in relation to blinding of participants and personnel, incomplete outcome data, selective reporting and other potential sources of bias (Figure 2a). On the other hand, less than 75% of the studies showed a low risk of bias in respect of blinding of outcome assessment, while less than 25% of the studies had a low risk of bias in terms of random sequence generation and allocation concealment. All the studies included in this review demonstrated either a low risk of bias or an unclear risk of bias in all the domains of assessment (Figure 2b).

### 5.2. Effects of Interventions

Based on the systematic review and meta-analysis, four distinct areas were identified: the effect of dietary fibre on gut microbiota; the role of dietary fibre on short-chain fatty acids; glycaemic control; and adverse events.

### 5.3. The Effect of Dietary Fibre on Gut Microbiota

The effects of dietary fibre on gut microbiota at the genus level are outlined in Table 3. According to Zhao et al. [13], the acetate-producing Bifidobacterium pseudocatenulatum was one of the most significantly promoted SCFA producers, and the enhancement of these positive responders reduced the producers of compounds, such as indole and hydrogen sulphide, which could be metabolically detrimental. There is evidence that patients with type 2 diabetes demonstrated intestinal dysbiosis based on the presence of increased levels of Prevotella copri [22]. However, following dietary intervention with functional foods, there was a significant modification of the faecal microbiota compared with control diet through the promotion of alpha diversity and an increased abundance of specific bacteria, independently of antidiabetic drugs [22].

However, Reimer et al. [31] found that the abundance of Lactobacillus spp. was greater in the control group, and although Faecalibacterium prausnitzii increased in both the dietary fibre and control groups, it was more profound in the control group (*p* = 0.038). Furthermore, there was a higher level for Collinsella spp. in the dietary fibre group compared with control. However, there were no significant differences in alpha or beta diversity [31]. According to Birkeland et al. [29], in the dietary fibre group, there was moderate changes in the faecal microbiota composition (1.5%, *p* = 0.045), and the effect was most prominent on operational taxonomic units (OTUs) of Bifidobacterium adolescentis, followed by OTUs of Bacteroides.

There was no significant effect on total bacteria, Lactobacillus, Roseburia, Enteroacteriaceae, Clostridium leptum or Clostridium coccoides groups following prebiotic fibre treatment in the study conducted by Pedersen et al. [23].

**Figure 3:**
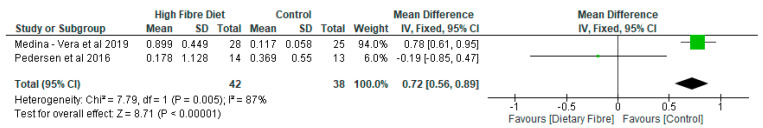
The effect of dietary fibre on Bifidobacterium (%).

The meta-analysis of Bifidobacterium, which was the only genus we were able to extract data for, involved two studies and 80 participants. There was significant difference (*p* < 0.01) in the relative abundance of Bifidobacterium with a mean difference of 0.72 (95% CI, 0.56, 0.89) between the dietary fibre group compared with placebo (Figure 3).

### 5.4. The Role of Dietary Fibre on Short-Chain Fatty Acids

In the study by Birkeland et al. [29], while the intervention (dietary fibre) group showed a significant increase in faecal concentrations of total SCFA (*p* = 0.04), acetic acid (*p* = 0.02), and propionic acid (*p* = 0.04) as compared to control group, there was no significant difference in relation to butyric acid between the treatments (*p* = 0.19) or on the overall microbial diversity [29].

In terms of the relationship between microbiota and the SCFA (acetic, propionic, butyric and valeric acid), a general trend was that acetic acid was positively related to operational taxonomic units (OTUs) that increased with the prebiotic fibre (Birkeland et al., 2020). On the other hand, the opposite trend was observed for the OTUs that declined with the prebiotic treatment, and the prebiotic affected OTUs of Bifidobacterium adolescentis were negatively related to butyric acid [29].

**Figure 4:**
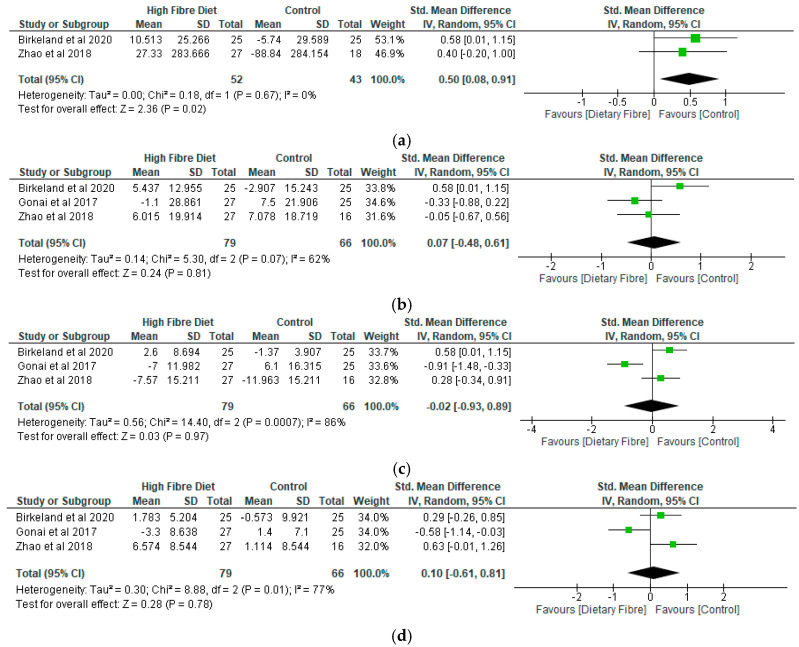
(**a**) The effect of dietary fibre on total short-chain fatty acids. (**b**) The effect of dietary fibre on acetic acid. (**c**) The effect of dietary fibre on propionic acid. (**d**) The effect of dietary fibre on butyric acid.

In relation to the meta-analysis for total SCFAs, two studies were included and involved 95 participants (Figure 4a). In contrast, 3 studies each and 145 participants, respectively, were included in the meta-analysis for acetic acid, propionic acid and butyric acid (Figure 4b–d). While there was a significant difference (*p* = 0.02) between the dietary fibre group and placebo with a standardised mean difference of 0.5 (95% CI, 0.08, 0.91) with respect to total SCFAs (Figure 4a), the differences were not significant (*p* > 0.05) in relation to acetic acid (Figure 4b), propionic acid (Figure 4c) and butyric acid (Figure 4d). Following sensitivity analysis, differences between the dietary fibre group and placebo were only significant (*p* = 0.04) for propionic acid and butyric acid when the study of Gona et al. [30] was removed, respectively, from the meta-analysis for each metabolite.

### 5.5. Glycaemic Control

According to Candela et al. [26], when patients with type 2 diabetes were randomised to follow the high-fibre diet or the control diet, there was significant reduction of fasting blood glucose and postprandial blood glucose in both diet groups. However, the difference was significantly higher for patients following the high-fibre diet compared with those following the control diet [26]. In the study by Soare et al. [27], the dietary fibre group showed a significantly greater reduction in glycated haemoglobin (*p* = 0.002) levels than the control group. Furthermore, Soare et al. [28] found that although both the dietary fibre and control diet groups maintained their benefits beyond the 21 days, the dietary fibre group resulted in greater improvement in glycaemic control following intensive monitoring over a 6-month period.

However, differences between the intervention and control groups were not significantly different with respect to glucose variables in the study conducted by Medina-Vera et al. [22]. Similarly, at 52 weeks, while patients with type 2 diabetes on intervention diet had a greater relative reduction in glycated haemoglobin from baseline (–3.19%) compared to control group (–0.57%) (*p* = 0.02); the differences between the groups were not statistically significant [31].

**Figure 5:**
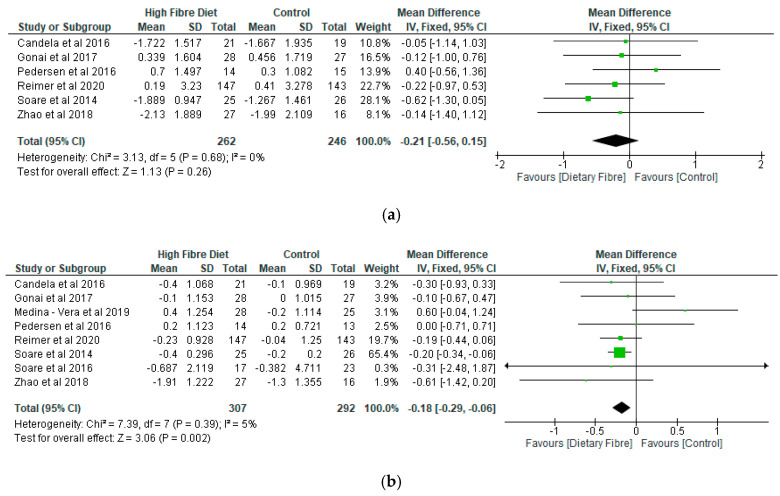

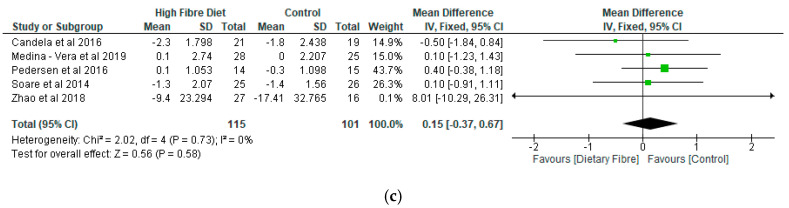
(**a**) The effect of dietary fibre on fasting blood glucose (mmols/L). (**b**) The effect of dietary fibre on glycated haemoglobin (%). (**c**) The effect of dietary fibre on homeostatic model assessment of insulin resistance (HOMA-IR).

In terms of the meta-analysis, while 6 studies were included for fasting blood glucose with 508 participants (Figure 5a), glycated haemoglobin had 8 studies with 599 participants (Figure 5b), and HOMA-IR had 5 studies with 216 participants (Figure 5c). However, there was only significant difference (*p* = 0.002) with respect to glycated haemoglobin with a mean difference of −0.18 (95% CI, −0.29, −0.06) between the dietary fibre group and placebo group (Figure 5b). Differences between the two groups were not significant (*p* > 0.05) in relation to fasting blood glucose (Figure 5a) and HOMA -IR (Figure 5c). Following a sensitivity analysis, the results for fasting blood glucose and HOMA-IR did not change. However, a difference between the dietary fibre group and placebo was not significant (*p* = 0.19) for glycated haemoglobin when the study of Soare et al. [27] was removed from the meta-analysis.

### 5.6. Adverse Events

There were no adverse side effects reported by the participants in the study by Pedersen et al. [23]. Furthermore, Gonai et al. [30] observed that galacto-oligosaccharides were well-tolerated, and no participant reported any severe adverse events. In the study by Reimer et al. [31], there were no significant differences (*p* > 0.05) between the two groups in subjects who reported adverse events (Table 4).

## 6. Discussion

The results of this systematic review and meta-analysis have shown that there were significant improvements (*p* < 0.05) in glycated haemoglobin, total SCFAs and the relative abundance of Bifidobacterium in the dietary fibre group compared with the control group. In contrast, differences between the two groups were not statistically significant (*p* > 0.05) in relation to fasting blood glucose, HOMA-IR, acetic acid, propionic acid, butyric acid and adverse events.

Some of the findings of this review would appear to confirm the results of an earlier systematic review by Houghton et al. [17] which assessed the effectiveness of dietary intervention on gut microbiota in adults with type 2 diabetes. Houghton et al. [17] also found significant improvement in glycated haemoglobin in the intervention group compared to control, but found no significant differences (*p* > 0.05) between the two groups in relation to fasting blood glucose, HOMA-IR and in the relative abundance of Bifidobacterium. The difference between this current review and the review by Houghton et al. [17] with respect to Bifidobacterium may be due to the type of dietary intervention, which was primarily dietary fibre in our review. Houghton et al. [17] observed that there were significant changes in the gut microbiota at the other taxonomic levels including phylum, family, genus and species. Fallucca et al. [32] have also reported that the Ma-Pi 2 diet has resulted in significant improvements in metabolic control, including fasting blood glucose and glycosylated haemoglobin in patients with type 2 diabetes in different continents.

Although several studies have reported the association between dysbiosis and type 2 diabetes, the results are often varied and inconsistent [6,33]. Dysbiosis has been reported to be positively associated with plasma glucose levels and the occurrence of type 2 diabetes [34]. It has been found that dysbiosis in the gut microbiota in patients with type 2 diabetes is characterised by a reduction in biodiversity [15,17]. Ebrahimzadeh Leylabadlo et al. [6] have also noted the findings of previous studies and demonstrated that type 2 diabetes was found to be associated with a reduction in the proportion of Firmicutes. Furthermore, the microbiome is characterized by the reduction of several butyrate-producing bacterial species including Clostridium, Eubacterium rectale, Faecalibacterium prausnitzii, Roseburia intestinalis, Roseburia inulinivorans and an enrichment of opportunistic pathogens [6]. The genera of Bifidobacterium, Bacteroides, Faecalibacterium, Akkermansia and Roseburia have been negatively associated with type 2 diabetes, while the genera of Ruminococcus, Fusobacterium, and Blautia have been positively associated with type 2 diabetes [35].

The mechanism by which changes in the community of gut microbiota modulates metabolic control is still evolving. However, a number of possible mechanisms have been documented including altering levels of glucagon-like peptide-1, lipopolysaccharides, inflammation and SCFAs [17]. The role of gut microbiota in type 2 diabetes involves microbial dysbiosis which harms the integrity of the intestinal wall and allows the translocation of bacteria, lipopolysaccharides, a metabolic endotoxemia from the gut lumen to the systemic circulation [2,36]. Furthermore, the endotoxemia causes low-grade inflammation, autoimmunity, and oxidative stress, which may lead to beta cell destruction or insulin resistance [2]. In other words, changes in the profile of gut microbiota cause gut permeability and loss of energy homeostasis, which leads to endotoxemia, low-grade inflammation, hyperglycaemia, hyperlipidaemia, obesity and insulin resistance [2,36]. Factors such as ethnicity, environment and socio-economic variables may influence the community of gut microbiota in terms of their abundance and diversity [15]. Other factors such as the use of broad spectrum antibiotics and changes in the quality of the diet including reduction in dietary fibre consumption could lead to disequilibrium in the community of gut microbiota [36]. Therefore, dysbiosis can elicit increased inflammatory activation through an increase in immune response to lipopolysaccharides, and this process contributes to the development of insulin resistance and type 2 diabetes [36].

The gut microbiota has been reported to influence energy levels, blood glucose and the effectiveness of pharmacological interventions [17]. While most of the insoluble fibres (e.g., cellulose and hemicellulose) are not fully digested by the gut bacteria and thus have a faecal bulking effect, most soluble fibres are fermented by the gut bacteria to produce metabolites, including SCFAs [7]. However, resistant oligosaccharides and most soluble NSPs are viscous and are able to form a gel structure in the intestinal tract, which can delay absorption of glucose and lipids, thus regulating post-prandial metabolism [7]. The gut microbiota and diet are two very useful components that keep the integrity of the gut intact and the production of intestinal mucus [7]. Thus, reduced intake of dietary fibre can lead to a reduction in the mucus layer and increase the risk of infection and the development of chronic inflammatory disease [7].

In the study by Candela et al. [26], which highlighted microbiota dysbiosis in patients with type 2 diabetes in contrast to healthy subjects, both diets were found to be effective in modulating gut microbiome dysbiosis in patients with type 2, leading to a bloom in ecosystem diversity in health-promoting SCFA producers, such as Faecalibacterium, Roseburia, Lachnospira, Bacteroides and Akkermansia. However, it was the high-fibre diet and not the control diet that was effective in counteracting the increase in possible pro-inflammatory groups, such as Collinsella and Streptococcus in the gut ecosystem and showing the potential to reverse pro-inflammatory dysbiosis in patients with type 2 diabetes, and possibly explaining the greater efficacy in improving the metabolic control [26].

In the current review, Gonai et al. [30] found that galacto-oligosaccharides (GOS) can ameliorate dysbiosis in patients with type 2 diabetes, and the long-term use of GOS may be an effective strategy for managing this condition. For example, while the abundance of Bifidobacteriaceae and the diversity of intestinal microbiota were significantly lower in patients with diabetes than in healthy subjects, Bifidobacteriaceae was significantly restored in patients with diabetes after consumption of GOS, although glucose tolerance did not improve during the period [30]. With respect to the dietary fibre, there was significant relationship between changes in gut microbiota components and changes in biochemical parameters [26]. It is possible that the promotion of SCFA producers in greater diversity and abundance by dietary fibre in this review led to improvement in glycated haemoglobin, partly due to increased glucagon-like peptide-1 (GLP-1) production [13]. In addition, Bifidobacterium lactis has been reported to increase glycogen synthesis, decrease expression of hepatic gluconeogenesis genes, improve translocation of glucose transport-4 and promote glucose uptake [35]. It is also possible that the reduction in body weight of participants in the intervention group due to the higher dietary fibre content of the diet [28] compared with control may have contributed to the observed improvement in glycated haemoglobin [31]. For example, in the study by Reimer et al. [31], at 52 weeks, only the high-fibre group had a significant decrease in body weight. In addition, Zhao et al. [13] and Soare et al. [27,28], found that the high-fibre group showed greater reduction in body weight than the control group. Weight loss ranging from 5–10% has been reported to improve glycated haemoglobin and obesity-related metabolic risk factors [31,37,38], while weight gain leads to deterioration in glycated haemoglobin levels [31,39]. A number of studies have also demonstrated that the consumption of a soluble, viscous fibre supplement can improve glycaemic control, including glycated haemoglobin in patients with type 2 diabetes [40,41,42].

The physico-chemical properties of dietary fibre are significant in influencing the function of the gastrointestinal (GI) tract including digestion, nutrient bio-accessibility, microbial fermentation and glycaemic control [43]. In addition, dietary fibre can have effect on the GI transit time and increased digesta viscosity, which can affect the flow of food [40,43]. The relative amounts and proportions of the components of dietary fibre such as cellulose, hemicellulose, pectin, lignin and water vary depending on the botanical source and the maturity of the plant tissue [43]. These properties have a significant effect on the physiology of digestion and gut function, including nutrient bio-accessibility, rate of gastric emptying and transit time, inhibition of flow, mixing efficiency of digesta, effects of gut microbiota and glycaemic control [43,44,45].

It would appear that a number of mechanisms are involved in the physiological effects of dietary fibre on macronutrient digestion, and the mechanism that predominates may be determined by a range of factors, including polysaccharide composition, the physical state of the fibre source, whether the fibre has been processed and the presence of other variables in foods such as lipids, which can also influence gut function [43].

Dietary fibres that are water soluble have been shown to reduce fasting plasma cholesterol in human subjects by modifying bile salt metabolism [43,46]. Soluble fibres have also been reported to lower plasma cholesterol levels by direct binding of polysaccharides to bile salts [43,47]. Another process of improving glycaemic control with a soluble fibre supplement is by significantly increasing the viscosity of chyme [40,48]. The increase in viscosity slows the interaction of enzymes and the nutrients, and this slows the breakdown of complex nutrients into absorbable forms and slows the absorption of glucose [40,47].

However, a major mechanism that has been identified in the control of blood glucose in patients with type 2 diabetes is the role of dietary fibre in physically encapsulating/entrapping nutrients, thus slowing the rate of digestion of plant tissues and reducing the rise in blood glucose [43,49]. The structural integrity of plant tissue can also be affected by food processing, and this can influence bio-accessibility and digestion [43]. For example, it has been reported that starch that is encapsulated with a leguminous cell is protected from digestion in the small intestine and produces a low glucose response compared to non-encapsulated starch [43].

The ranking of different carbohydrate foods can also be based on their glycaemic index [50,51]. Therefore, foods that have low glycaemic index, such as lentils, beans and oat, provide gradual supply of glucose to the blood and thus ensure a more sustained insulin release [50]. Soluble fibre is closely related to the concept of glycaemic index by delaying the absorption of dietary carbohydrates due to its viscous and gel-forming properties, thus reducing postprandial glucose excursions [52].

## 7. Limitation of the Review

Although nine studies were included in the overall meta-analysis, the studies included in the meta-analysis for SCFAs and gut microbiota were no more than three and two studies, respectively, and this could limit the wider application of the findings. Therefore, more studies are required in this area of research. In addition, despite the use of the random effects model in the analysis of short-chain fatty acids, the high heterogeneity of the studies included may have also affected the results of the meta-analysis.

## 8. Conclusions

This systematic review and meta-analysis have demonstrated that dietary fibre can significantly improve (*p* < 0.05) the relative abundance of Bifidobacterium, total SCFAs and glycated haemoglobin. However, dietary fibre did not appear to have a significant effect (*p* > 0.05) on fasting blood glucose, HOMA-IR, acetic acid, propionic acid, butyric acid and adverse events.

## Figures and Tables

**Table 1:** Search Terms and Search Strategy.

Patient/Population	Intervention	Outcome (Primary)	Study Designs	Combining Search Terms
Patients with diabetes	Dietary fibre	Gut microbiota	Randomised controlled trial	
Patients with diabetes OR type 2 diabetes OR Diabetes OR Diabetes complications OR diabetes mellitus, type 2 OR diabetes mellitus	Dietary fibre OR Dietary supplements OR Dietary carbohydrate OR Polysaccharide OR Wheat bran OR Fibre OR Supplement OR Prebiotics	Microbiota OR Gut microbiota OR Gastrointestinal microbiota OR Microbiome	#1 Randomised controlled trial OR controlled clinical trial OR randomized OR placebo OR drug therapy OR randomly OR trial OR groups #2 “Animals” NOT “Humans” #3 #1 NOT #2	Column 1 AND Column 2 AND Column 3 AND Column 4

**Table 2:** The description and characteristics of included studies.

Citation/Country of Study	Study Type	Sample Details	Mean Age (Years)	Aim	Interventions	Results
Birkeland et al. [29]. Norway	Randomised, placebo controlled, double-blind, cross-over study	*n* = 25	63.1 (41–73)	To evaluate the prebiotic effect of inulin-type fructans on faecal microbiota and SCFA in patients with T2D.	Inulin-type fructans (a mixture of oligofructose and inulin) versus placebo (maltodextrin). A 4-week washout separated the 6 weeks of treatment.	A daily supplement of inulin-type fructans induced a moderate, but significant increase in faecal levels of bifidobacteria, total SCFA, acetic acid and propionic acid in patients with T2D.
Candela et al. [26]. Italy	Open-label randomised controlled trial	Ma-Pi 2 diet: *n* = 21; Control diet: *n* = 19.	66	To explore the potential of two different energy-restricted dietary approaches—the fibre-rich macrobiotic Ma-Pi 2 diet or a control diet recommended by Italian professional societies for T2D treatment—to correct gut microbiota dysbiosis in T2D patients.	Fibre-rich macrobiotic Ma-Pi 2 diet versus control diet. 21 days of treatment.	The Ma-Pi 2 diet was associated with a greater reduction in FBG. Body weight changes (mean ± SD) kg: Ma-Pi 2 diet: −5.6 ± −1.0 Control diet: −2.7 ± −0.6
Gonai et al. [30]. Japan	Randomized controlled, double-blind study	GOS: *n* = 27; Placebo: *n* = 25.	GOS: 55 ± 11 Placebo: 54 ± 12.	To assess the effects of GOS on glycaemic control and gut microbiotas and metabolites in patients with T2D.	Galacto-oligosaccharide (GOS) versus placebo (maltodextrin). Four weeks of treatment.	GOS restored the abundance of Bifidobacteriaceae. However, GOS did not have a significant effect on glucose tolerance.
Medina-Vera et al. [22]. Mexico	Single-centre, placebo-controlled, randomised double-blind	T2D: *n* = 81 (randomised: 9 subjects from each group discontinued study) final group numbers analysed: DF: *n* = 28 Placebo: *n* = 25	DP: 50.4 ± 8.7 Placebo: 49.8 ± 10.6	To study the effects of a functional food-based dietary intervention on faecal microbiota and biochemical parameters in patients with T2D.	A dietary portfolio (DP) versus placebo. A 3-month treatment period.	DP consumption stimulated the abundance of Bifidobacterium longum shown to improve insulin sensitivity. There were no significant differences in the levels of glucose between groups. Patients with T2D following the DP showed significant reductions in specific biochemical parameters compared with the placebo group: AUCs for glucose.
Pedersen et al. [23]. UK	Randomised, double-blind, placebo-controlled parallel study	GOS: *n* = 14; Placebo: *n* = 15.	GOS: 56.7 ± 1.6; Placebo: 58.1 ± 1.7.	To compare the effects of prebiotic supplementation with placebo treatment for 12 weeks on glucose control, intestinal permeability, intestinal bacterial composition, and endotoxaemia in patients with T2D.	Galacto-oligosaccharide (GOS) versus placebo (maltodextrin): 12 weeks of treatment.	Prebiotic fibre supplementation had no significant effects on clinical outcomes or bacterial abundances compared with placebo. Body weight changes (mean ± SEM) kg: GOS: 0.6 ± 0.1 Placebo: 0.1 ± 0
Reimer et al. [31]. Canada	Placebo-controlled, double-blind, randomised controlled study	PGX^®^: *n* = 147 Placebo: *n* = 143.	PGX^®^: 56.2 ± 8.6 Placebo: 53.4 ± 9.9.	To examine the adjunct effect of the soluble viscous fibre PolyGlycopleX^®^ (PGX^®^) on glycaemic control in adults with T2D.	PGX^®^ versus placebo 52 weeks of treatment.	The butyrate producer (Roseburia) was significantly increased in the PGX^®^ group. Adding PGX^®^ to a weight management program for individuals with T2D provides a sustained reduction in HbA1c compared to placebo. Body weight changes: mean (95% CI) kg PGX^®^: −3.87 (−1.75 to −6.0) Placebo: −1.62 (0.56 to −3.80)
Soare et al. [27]. Italy	Randomized controlled, open-label trial	Ma-Pi 2 diet: *n* = 25; Control diet: *n* = 26	Ma-Pi 2 diet: 67 ± 8.163 Control diet: 65 ± 7.284	To evaluate the effect of different dietary approaches—the macrobiotic Ma-Pi 2 diet compared with standard diets recommended for patients with T2D.	Fibre-rich macrobiotic Ma-Pi 2 diet versus control diet: 21 days of treatment.	There was significantly greater reduction in fasting blood glucose, HbA1c, and insulin resistance in those patients receiving the Ma-Pi 2 diet compared with those in the control diet group. Body weight changes (mean ± SD) kg Ma-Pi 2 diet: −4.9 ± 0.4 Control diet: −3.97 ± 0.08
Soare et al. [28]. Italy	Randomized controlled, open-label trial. 6-month follow-up study	Ma-Pi 2 diet: *n* = 17 Control diet: *n* = 23.	Ma-Pi 2 diet: 65 ± 8.89 Control diet: 64 ± 8.15	To investigate whether the benefits of the original 21-day intensive dietary interventions extended beyond the original MADIAB trial duration and into everyday life.	Fibre-rich macrobiotic Ma-Pi 2 diet versus control diet: 6 months of treatment.	The Ma-Pi diet was associated with a higher percentage reduction in HbA1c. The Ma-Pi diet resulted in greater improvement in glycaemic control. Body weight changes: median (1st–3rd quartile) kg Ma-Pi 2 diet: −1.46 (−4.59; 0.78) Control diet: 0.72 (−2.4; 3.26)
Zhao et al. [13]. China	Randomized controlled trial, open-label, parallel-group study	High dietary fibre: *n* = 27, Control: *n* = 16.	High dietary fibre: 58.4 ± 6.2. Control: 59.7 ± 6.0;	To characterise the dynamics of the gut microbiota and its impact on glucose homeostasis in patients with T2D.	High dietary fibre versus control (usual care)	A select group of SCFA-producing strains was promoted by dietary fibres, and most other potential producers were either diminished or unchanged in patients with T2D. Body weight changes (mean ± SEM) kg: High dietary fibre: −2.99 ± −0.16 Control: −1.09 ± −0.13

Abbreviations: AUCs—areas under the curve; DP—dietary portfolio; FBG—fasting blood glucose; GOS—galacto-oligosaccharide; HbA1c—glycated haemoglobin; kg—kilogram; Ma-Pi 2—macrobiotic diet; PGX^®^—PolyGlycopleX^®^; T2D—type 2 diabetes; SCFAs—short-chain fatty acids.

**Table 3:** The effect of dietary fibre on gut microbiota at the genus level.

Citations	Bacteroides	Clostridium	Lactobacillus	Ruminococcus	Roseburia	Bifidobacterium
Birkeland et al. [29].	There was also a positive effect on operational taxonomic units of Bacteroides.	Not Applicable	Not Applicable	Not Applicable	Not Applicable	A bifidogenic effect was most prominent, with the highest positive effect on operational taxonomic units (OTUs) of Bifidobacterium adolescentis
Candela et al. [26].	Both diets increased the abundance of propionate and butyrate producers (i.e., Bacteroides)	Not Applicable	Not Applicable	Both diets consolidated a healthy-like abundance of Ruminococcus	Both diets consolidated a healthy-like abundance of Roseburia	Not Applicable
Gonai et al. [30].	Not Applicable	Not Applicable	Not Applicable	Levels of Ruminococcaceae were significantly lower after intake of GOS compared with the baseline	Not Applicable	Bifidobacteriaceae abundance was considerably increased by intake of GOS compared with the baseline.
Medina-Vera et al. [22].	Not Applicable	Not Applicable	Not Applicable	Not Applicable	Not Applicable	DP consumption stimulated the abundance of Bifidobacterium longum
Pedersen et al. [23].	Not Applicable	Prebiotic treatment had no significant effect on Clostridium leptum or Clostridium coccoides groups.	Prebiotic treatment had no significant effect on total bacteria, Lactobacillus	Not Applicable	Prebiotic treatment had no significant effect on Roseburia	Prebiotic treatment had no significant effect on Bifidobacterium or any of the other bacteria measured
Reimer et al. [31].	Not Applicable	Not Applicable	Not Applicable	Not Applicable	PGX^®^ significantly increased the relative abundance of Roseburia	Not Applicable
Soare et al. [27].	Not Applicable	Not Applicable	Not Applicable	Not Applicable	Not Applicable	Not Applicable
Soare et al. [28].	Not Applicable	Not Applicable	Not Applicable	Not Applicable	Not Applicable	Not Applicable
Zhao et al. [13].	Not Applicable	Not Applicable	Not Applicable	Not Applicable	Not Applicable	Bifidobacterium pseudocatenulatum was one of the most significantly promoted SCFA producers

Abbreviations: DP—dietary portfolio; GOS—galacto-oligosaccharide; PGX^®^—PolyGlycopleX^®^; SCFA—short-chain fatty acid.

**Table 4:** The most common adverse events reported over 52 weeks in participants allocated to PGX^®^ or placebo.

	PGX^®^ (*n* = 103)	Placebo (*n* = 104)
Total adverse events	580	525
Most common events:		
Diarrhoea/loose stool	150	61
Cold/flu-like symptoms	56	71
Abdominal bloating	35	29
Abdominal pain/cramps	35	37
Constipation	17	48
Headache/sinus pain	27	45

Abbreviations: PGX^®^—PolyGlycopleX^®^. Data from Reimer et al. [31].
